# Traditional Chinese Medicine for osteoporosis management: from molecular mechanisms to drug discovery

**DOI:** 10.1186/s13020-026-01353-9

**Published:** 2026-02-27

**Authors:** Shenyu Yan, Qiaohui Du, Lijun Zhao, Qing Liu, Meiling Wu, Ziqiao Xu, Jiangang Shen

**Affiliations:** 1 School of Chinese Medicine, Li Ka Shing Faculty of Medicine, The University of Hong Kong, 3 Sassoon Road, Pokfulam, Hong Kong, Hong Kong SAR, China; 2 Hainan Academy of Medical Sciences, Hainan Medical University, Hainan, China

**Keywords:** Osteoporosis, Traditional Chinese Medicine, Multi-target therapy, Bone remodeling, Drug discovery

## Abstract

**Abstract:**

Osteoporosis, a prevalent skeletal disorder, poses significant challenges in aging populations. Traditional Chinese Medicine (TCM) offers a multi-target approach to addressing its complex pathogenesis, which involves hormonal imbalance, oxidative stress, and inflammation. This review highlights the current progress in osteoporosis, focusing on the molecular mechanisms and therapeutic targets of relevant mechanisms, as well as the related cellular events and signaling pathways. Furthermore, the study reviews the clinical effectiveness of several representative TCM formulae, such as Xianling Gubao Capsule and Qing’e pill, which can reduce fracture risk by promoting bone formation and suppressing bone resorption. The study also discusses the therapeutic principles of the promising medicinal herbal compounds, such as icariin for enhancing osteogenesis, naringin for inhibiting osteoclastogenesis, and astragaloside IV for reducing cellular senescence. In summary, TCM's holistic approach offers a valuable strategy for managing osteoporosis, particularly for high-risk patients. Integrating traditional knowledge with modern science enables the development of safer, multi-target therapies addressing both bone quantity and quality. Continued research will facilitate evidence-based TCM interventions for global osteoporosis care.

**Graphical Abstract:**

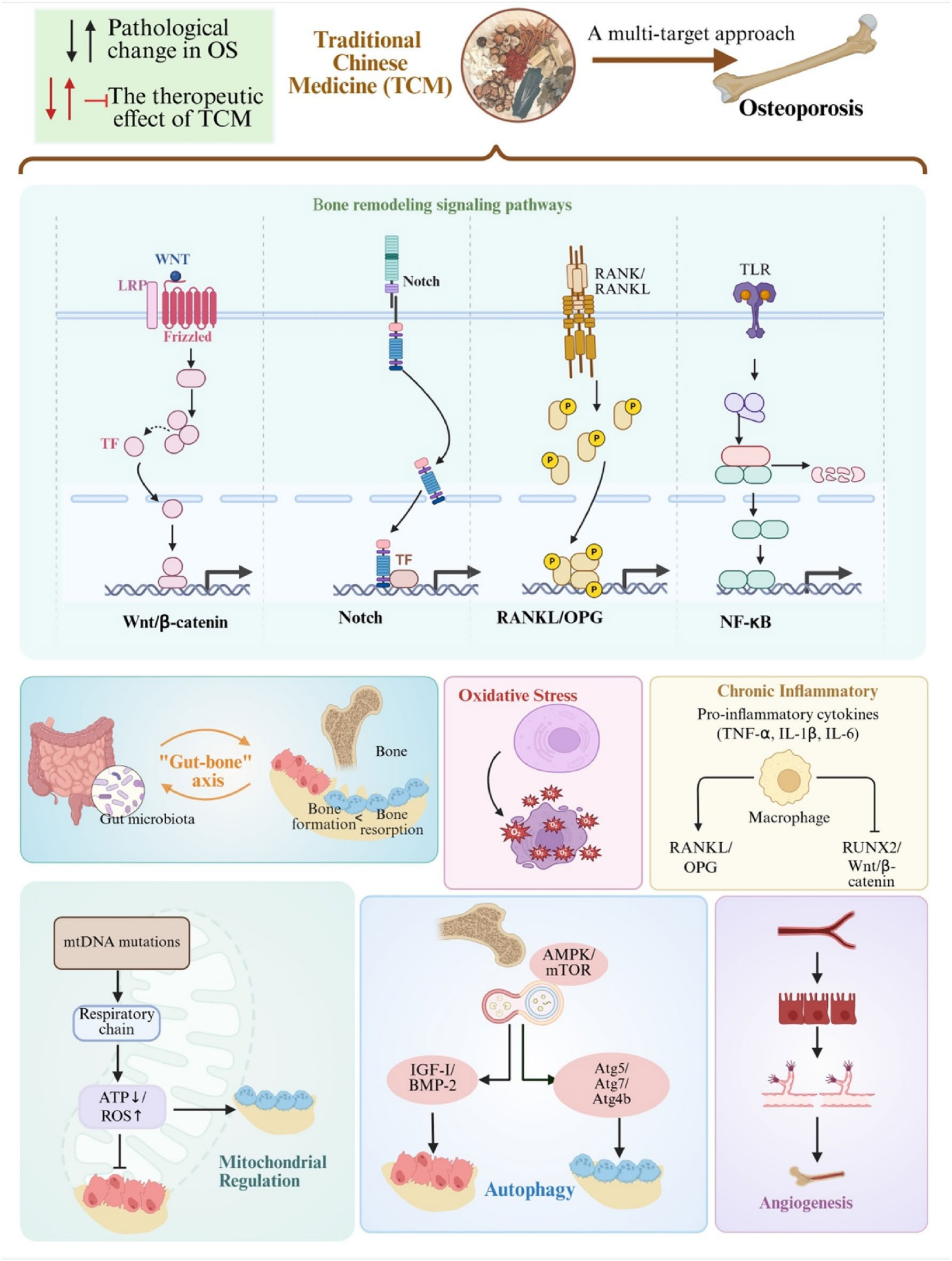

## Introduction

Osteoporosis is a systemic skeletal disorder characterized by low bone mineral density (BMD) and deterioration of bone microarchitecture, resulting in increased bone fragility and susceptibility to fracture [1, 2]. Etiologically, osteoporosis can be classified into primary, secondary, and idiopathic forms. Primary osteoporosis is the most common type due to the age-related bone loss or postmenopausal estrogen deficiency [3, 4]. While, the secondary osteoporosis results from specific medical conditions or medications that adversely affect bone metabolism, mechanistically involving inflammatory cytokines, glucocorticoid-induced suppression of osteoblast activity and promotion of osteoclast survival, hormone deficiencies, or drug-related interference with bone remodeling [4, 5]. Idiopathic osteoporosis is a rare form occurred in younger individuals without an identifiable cause [5]. Osteoporosis represents one of the major global public health concerns, affecting approximately 200 million people worldwide [1, 2]. According to the 2019 Global Burden of Disease, an estimated 178 million new fragility fractures occurred globally, a number rising in proportion to aging populations [1, 2]. Osteoporosis disproportionately impacts older adults, with the highest prevalence and fracture incidence observed in postmenopausal women, particularly those over 80 years of age [6]. While women most frequently sustain fractures of the wrist, hip, or vertebrae, men experience the same fracture types at significantly lower rates [7].

According to World Health Organization criteria, osteoporosis is defined as a BMD T-score of -2.5 standard deviations or below at the spine or hip in postmenopausal women [8]. Disease progression is influenced by multiple factors, including hormonal imbalances, nutritional deficiencies, smoking, excessive alcohol consumption, and certain medications [8]. Thus, diagnosis of osteoporosis primarily relies on BMD assessment, which, in combination with clinical risk factors, estimates individual fracture risk [1]. The current gold standard utilizes dual-energy X-ray absorptiometry, with computed tomography as an alternative [7].

Effective management and prognosis depend on improving musculoskeletal strength and physical fitness, which influence bone resorption, calcification, and hormonal metabolism. Treatment strategies should consider both lifestyle modifications and hormonal homeostasis to develop personalized therapeutic approaches. Current pharmacological regimens primarily function by either inhibiting bone resorption with anti-resorptive agents like bisphosphonates or stimulating bone formation with osteoanabolic agents like teriparatide [9]. However, their utility is often limited by adverse effects, including musculoskeletal discomfort, bone pain, and potential long-term risks [10, 11]. Therefore, while developing more effective therapies remains crucial, identifying treatments with minimal side effects and a focus on enhancing patients' quality of life is equally vital for the comprehensive management of osteoporosis.

Traditional Chinese Medicine (TCM) has shown promising effects on osteoporosis by targeting multiple signaling pathways with well-organized herbal formulae [12–14]. The use of herbs for bone health has been documented in ancient classical textbooks, including *Shen Nong’s Materia Medica* and the *Compendium of Materia Medica*, with several historically noted components now validated by modern research for their osteoprotective benefits [15]. In clinical practice, TCM is frequently used either as a monotherapy or as an integrated adjunct to conventional Western medicine for osteoporosis management. A pharmacological study shows promising effectiveness in promoting bone mineralization and angiogenesis, stimulating osteogenesis for bone formation, and inhibiting osteoclastogenesis for bone resorption [16]. The integration of TCM strategy with modern conventional therapeutic technologies offers advanced approaches to osteoporosis care, improving prognosis and outcomes. TCM approaches exert characteristic multi-targets, synergistic mechanisms, and favorable safety profiles, indicating distinct complementary advantages in the comprehensive management of osteoporosis. In this extensive review, we discuss the primary pathogenesis of osteoporosis and the therapeutic applications of TCM formulae, as well as the active compounds and extracts of single herbs, aiming to explore their principal mechanisms and provide a foundation for future investigations.

## Pharmacological mechanisms and molecular targets for osteoporosis in TCM therapy

Osteoporosis is characterized by reduced BMD and deterioration of bone microarchitecture, which leads to increased bone fragility and fracture risk [17]. The osteoporotic pathogenesis is defined by an imbalance between bone resorption and formation, driven primarily by osteoclast overactivation, osteoblast dysfunction, and impaired osteocyte autophagy [18–21]. This remodeling process is regulated by multiple signaling pathways, including Wnt/β-catenin, Notch, RANKL/OPG, and NF-κB, which are representative signaling pathways that regulate bone remodeling [22]. Oxidative stress, chronic inflammation, mitochondrial dysregulation, and impaired angiogenesis are critical factors that exacerbate bone loss [23–25]. Emerging evidence also underscores the significant role of the gut-bone axis in disease progression. Therefore, osteoporotic pathogenesis involves a complex interplay of cellular and molecular events. In the following section, we review current progress in understanding how TCM targets these pathological processes, as summarized in Tables 1 and 2.

**Table 1:** Activated compounds targeted mechanisms in osteoporosis

Activate compounds	Structure	Origin(Chinese herbs)	Model	Description of Mechanism	References
Flavonoids		Drynariae Rhizoma	OVX rats	Activate the Wnt3a/β-catenin pathway	[36]
Naringin		*Citrus fruit*	OVX mouse mode Mc3T3-E1 cells	Activate Wnt/β-catenin signaling pathway; Modulated Jak2/STAT3 signaling; Inhibited NF-κB signaling; Reduced osteoclast; Activated mitochondrial apoptosis	[37, 65, 124]
Bergamottin		Citrus fruits	OVX model	Activate Wnt/β-catenin signaling pathway	[38]
Icariin		Epimedium-derived flavonoids	human bone mesenchymal stem cell	Activate Wnt/β-catenin signaling pathway; gut microbiota	[39, 82]
Artesunate		Artemisinin	bone marrow mesenchymal stem cells; OVX model	Inhibit the NF-κB and Notch1/Hes1 signaling pathways	[46]
Castanoside A		Cistanche deserticola	OVX model	Modulate of RANKL/RANKL/TRAF6-mediated NF-kB and PI3K/AKT signaling	[52, 53]
Echinacoside		Cistanche deserticola	OVX model	Modulate of RANKL/RANKL/TRAF6-mediated NF-kB and PI3K/AKT signaling	[52, 53]
Quercetin		*Cuscuta chinensis* Lam	OVX model	Inhibited RANKL; Activated p38 and AKT signaling pathway	[55, 56]
Kaempferol		*Cuscuta chinensis* Lam	Molecular docking	Modulate RANKL	[56]
Astragalin		*Cuscuta chinensis* Lam	Cell model	Regulated ROS and MAPK signaling pathway	[54]
6-gingerol		Rhizome of ginger	Cell model	Down regulate of RANKL expression	[57]
Curcumin		Turmeric root; Curcuma longa	OVX model; Clinical studies	Inhibit the RANKL-RANK-TRAF6 signaling; Reduced mitochondrial ROS; Enhanced complex III and coenzyme Q activity	[58, 122]
Morusin		Morus alba	OVX model	Decrease RANKL-induced osteoclastogenesis	[62]
Dihydrotanshinone I		Salvia miltiorrhiza	Cell model	Modulate critical signaling pathway including NF-κB, ERK, and calcium ion signaling	[69]
Casticin		*Vitex agnus-castus*	Cell model	Inhibited NF-κB/MAPK pathway	[70]
α-Mangostin		Mangosteen	Cell model	Disrupt the activation of NF-κB/mitogen-activated protein kinase signaling pathways	[71]
Resveratrol		*Polygonum cuspidatum*	OVX rats	Attenuate Nox4/NF-κB signaling pathway	[72]
Astragaloside IV		*Astragalus membranes*	d-galactose-induced model	Suppressed the STING/NF-κB pathway; Attenuate of inflammation	[66]
Luteolin		Honeysuckle; perilla	OVX model	Attenuated ROS accumulation and mitochondrial dysfunction	[95]
Cirsilineol		Artemisia plants	OVX model	Inhibited osteoclast activation; Regulated NF-κb/ERK/p38 signaling pathways	[67]
Roburic acid		RAdix Gentianae Macrophyllae	OVX model	Modulated the RANKL-associated ERK/NF-κB/NFATc1 pathway	[68]
Angelicin		Psoralea	OVX model	Decreased ROS; Inhibited oxidative stress levels and osteoclast formation	[96]
Icaritin		Icariin	Ferric ammonium citrate -induced model	Antioxidant; Osteoprotective effects	[97]
Irisolidone		Pueraria lobata flowers	OVX model	Stimulate activity via the AMPK-ULK1-autophagy axis	[114]
Arbutin		*Bearberry plants*	Glucocorticoid dexamethasone-induced	Downregulate of osteogenic gene; reduced autophagic marker expression; Decreased autophagic puncta formation	[115]
Notoginsenoside R1		Panax notoginseng	MC3T3-E1 cells	Attenuated OS-induced mitochondrial damage	[123]
Vitexin		Panax notoginseng saponins	OVX model	Promote trabecular bone repair; Enhance osteogenic; Vascular restoration	[137]
Ginsenoside Rg1		Panax ginseng	Cell model; Goto-kakizaki rat model	Promote angiogenesis and osteointegration coupling	[138]
Eleutheroside E		Acanthopanax senticosus	Network pharmacology; OVX model	Regulated gut microbiota	[87]

**Table 2:** Chinese herbs targeted mechanism in osteoporosis

Herbs	Model	Mechanism	References
Gu ling pian	Osteoblast cell model	Promote osteoblast differentiation; Regulated OPG/RANKL expression	[51]
Drynaria fortunei	Clinical studied	Influenced NLRP3 inflammasome;	[61]
Antrodia cinnamomea	OVX model	Inhibited RANKL-induced osteoclast formation	[63]
Cuscuta chinensis Lam	Meta-analysis	Antiosteoporotic; Antioxidant; Anti-aging	[64]
Moringa oleifera leaf	OVX model	Improved gut microbiota composition; Increased the expression of Occludin and Claudin-1 protein in the duodenum	[81]
Achyranthis bidentatae	Dexamethasone zebrafish model	Regulated redox balance; Influenced mitochondrial integrity	[98]
Rhizoma Drynariae	Cell model; OVX model	Improved BMD; Regulated immune-inflammatory pathway	[104, 105]
Jintiange	Cell model; meta-analysis	Inhibited apoptosis; Regulated BMP/ Wnt/β-catenin pathway /NF-κB signaling;	[35, 116]

### Targets bone remodeling signaling pathways

**Fig. 1:**
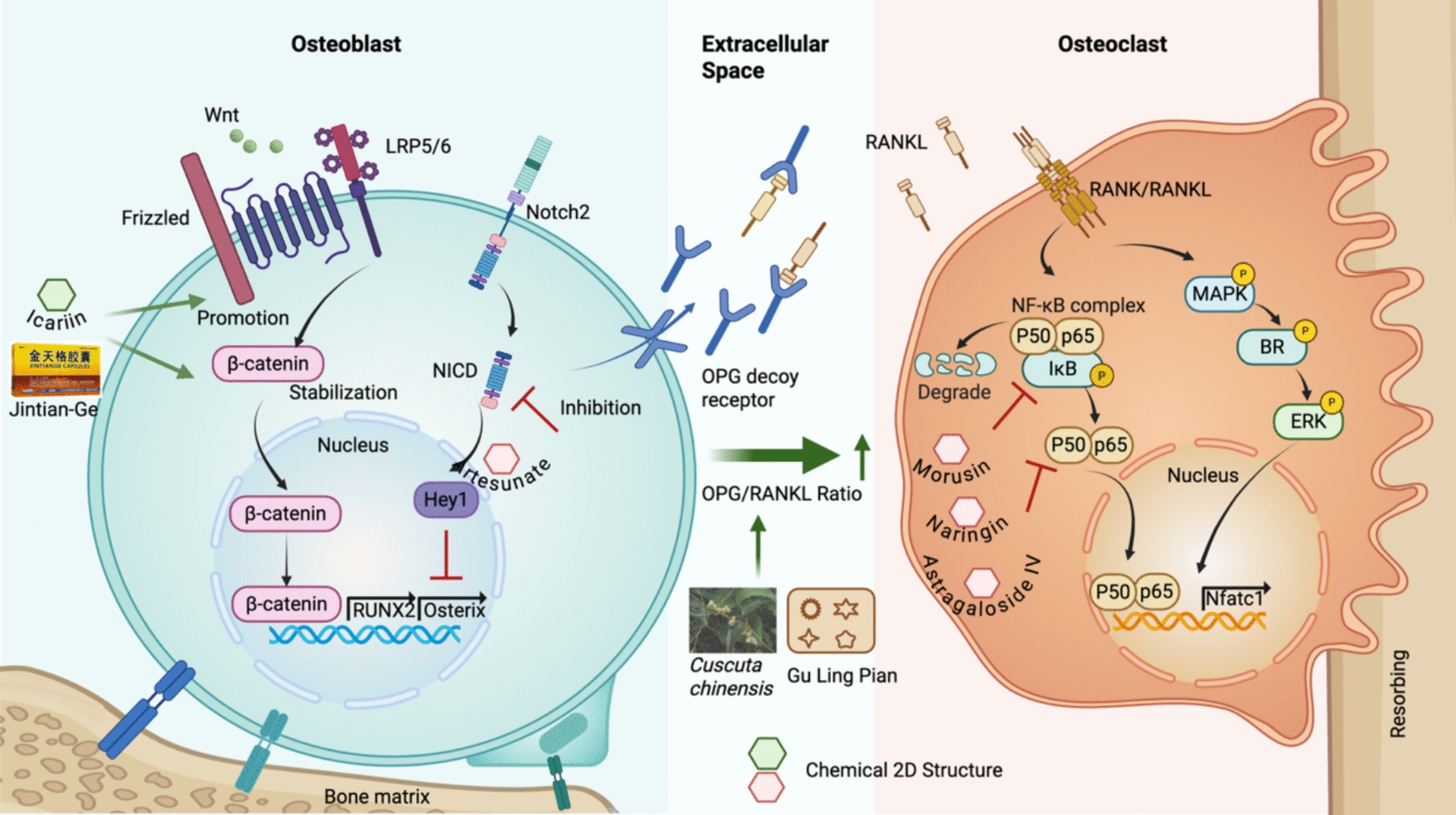
Molecular targets regulating osteoblast and osteoclast in osteoporotic pathogenesis and current understanding of therapeutic mechanisms of medicinal plants and their active compounds. Blue: Osteoblast; Yellow: Osteoclast

The pathogenesis of osteoporosis is fundamentally driven by the disruption of bone homeostasis, which relies on the tightly regulated balance between osteoblast-mediated bone formation and osteoclast-mediated bone resorption [22, 26, 27]. This balance is orchestrated by a complex network of signaling pathways, including the Wnt/β-catenin, RANKL/OPG, Notch, and NF-κB pathways, whose dysregulation leads to impaired osteoblast function, excessive osteoclast activation, and ultimately, progressive bone loss (Fig. 1).

#### Wnt/β-catenin signaling pathway

Wnt/β-catenin signaling is pivotal in osteoblast differentiation and bone formation via mediating β-catenin nuclear translocation and activating RUNX2 and Osterix [28, 29]. Wnt signaling also indirectly regulates osteoclast differentiation by inhibiting RANKL expression and promoting OPG secretion, thereby reducing the RANKL/OPG ratio [30, 31]. Conversely, non-canonical Wnt5a promotes osteoclast formation and bone resorption by activating the ROR2/JNK pathway and upregulating RANK expression [31, 32]. These findings underscore the dual regulatory role of Wnt signaling in bone remodeling and its therapeutic relevance for osteoporosis.

The activation of Wnt/β-catenin signaling pathway is one of the molecular targets involved in anti-osteoporotic effects of TCM therapy and related active components. Qiangquyin is a traditional Chinese medicine prescription for postmenopausal osteoporosis. A modified formula named N-QGY revealed to enhance osteogenesis in OVX rat models via exosome-mediated activation of the Notum/Wnt/β-catenin axis and upregulation of key bone formation markers [33]. Jin-Tian-Ge (JTG) is an artificial tiger bone powder prepared from skeletons of several farmed animals to substitute natural tiger bone for osteoporosis. JTG prevented bone loss and improved the bone microarchitecture in an OVX rat model in vivo*.* Meanwhile, JTG promoted the osteogenic differentiation of BMSCs in vitro through regulating BMP and Wnt/β-catenin pathways, and inhibited osteoclastogenesis by suppressing the NF-κB pathway [34]. A systematic review revealed the effects of JTG on relieving pain, improving BMD, and preventing fractures in primary osteoporosis patients [35]. Flavonoids from Rhizoma Drynariae exhibited to activate the Wnta/β-catenin pathway and modulates lipid metabolism, inflammatory factors, and immune responses [36]. Similarly, naringin and bergamottin inhibited osteoclastogenesis and promoted osteogenesis via affecting Wnt and JAK2/STAT3 pathways [37, 38]. Additionally, Icariin enhanced osteoblast proliferation, suppresses osteoclast activity, and improves bone microarchitecture through stimulating Wnt/β-catenin signaling pathway [39]. Thus, Wnt/β-catenin signaling pathway could be a therapeutic target for anti-osteoporosis.

#### Notch signaling

Notch signaling could be a key regulator in the process of osteoblast proliferation and differentiation. Notch2 deficiency has been shown to enhance osteogenesis and increase trabecular bone mass [40]. A reciprocal crosstalk exists between Notch and Wnt/β-catenin pathways: Wnt activation promotes osteoblast differentiation while suppressing Notch, whereas excessive Notch activity inhibits osteogenesis by downregulating Wnt signaling [41]. Notch signaling enhances Wnt-induced bone formation by upregulating OPG and inhibits osteoblast gene expression by suppressing Runx2 transcriptional activity through the downstream effector Hey1 [42]. The generation of osteoclasts depends on activating the transcription factor Nfatc1. Notch2 intracellular domain interacts with NF-κB to drive Nfatc1 transcription and osteoclast differentiation [43, 44].

Notch signaling presents a potential therapeutic target for osteoporosis. Jiangu granules, a commonly used TCM formula, improves bone metabolism in OVX rat models through the coordinated regulation of Notch and steroid hormone pathways [45]. Artesunate is a bioactive compound derived from Artemisia annua. Artesunate revealed to improve bone microarchitecture in OVX rats by inhibiting the Notch1/Hes1 signaling axis and promoting osteogenic differentiation [46]. Therefore, Notch signaling could be a promising therapeutic target to inducing osteogenesis and increasing trabecular bone mass.

#### RANKL/OPG signaling pathway

The imbalance of the RANKL/OPG axis also contributes to excessive bone resorption and bone loss in the pathogenesis of osteoporosis [47]. RANKL is secreted by osteoblasts, bone marrow stromal cells, and activated T cells. RANKL binds to the RANK receptor on the surface of osteoclast precursors, activating downstream signaling pathways such as NF-κB and MAPK [48]. OPG, a soluble decoy receptor secreted by osteoblasts, competitively binds to RANKL, blocking its interaction with RANK, thereby inhibiting osteoclast formation and maintaining bone homeostasis [49]. The disruption of RANKL and OPG enhances osteoclast activity, accelerates bone resorption, and damages bone microstructures [50]. Gu Ling Pian, a TCM kidney-tonifying herb, revealed to prevent osteoporosis by regulating the OPG/RANKL and p38 MAPK signaling pathways [51]. Cistanche deserticola contains castanoside A and echinacoside, which inhibits osteoclast differentiation and bone resorption via RANKL/RANK/TRAF6 signaling [52] [53]. Favonoids from Cuscuta chinensis including quercetin, kaempferol, and astragalin alleviated bone resorption and restored osteogenic markers by modulating the RANKL/OPG axis [54, 55] [56]. Additionally, ginger-derived compounds such as curcumin and 6-shogaol also show promising effects on hormone-induced osteoporosis models [57, 58].

#### NF-κB signaling pathway

The NF-κB signaling pathway is a critical downstream effector of the RANKL-RANK interaction and a key convergence point for inflammatory stimuli in bone metabolism [59]. Its inhibition effectively blocks RANKL-induced osteoclast formation, highlighting its significance in the progression of osteoporosis [60]. Accumulating evidence indicates that numerous TCM compounds and formulations exert anti-osteoporotic effects by modulating this pathway. For instance, *Drynaria fortunei* significantly increased bone mineral density while reducing serum NF-κB levels in elderly patients with postmenopausal osteoporosis [61]. In a preclinical study, Morusin, derived from Morus australis, inhibited RANKL-induced NF-κB and MAPK signaling, thereby protecting against bone loss in murine models [62]. Similarly, extracts from *Antrodia cinnamomea* and *Cuscuta chinensis* suppressed osteoclastogenesis via the NFATc1 and NF-κB/IκBα pathways [63, 64]. Naringin, a citrus flavonoid, potently inhibited NF-κB signaling by reducing p65 nuclear translocation by 60% in RANKL-stimulated RAW264.7 cells [65] [37]. Astragaloside IV, a representative compound from *Astragalus membranaceus*, inhibited macrophage senescence and M1 polarization, alleviated mitochondrial dysfunction, and promoted M2 polarization via suppressing the STING/NF-κB pathway in a senile osteoporosis models [66]. Cirsilineol, an active constituent of Vestita Wall, inhibited RANKL-induced osteoclast activity and ovariectomy-induced bone loss via NF-kappab/ERK/p38 signaling pathways [67]. Roburic acid from *Radix Gentianae Macrophyllae* attenuated osteoclastogenesis and bone resorption by targeting RANKL-induced intracellular signaling pathways [68]. Moreover, Dihydrotanshinone I, derived from *Salvia miltiorrhiza*, inhibited osteoclastogenesis by suppressing the NF-κB/ERK/NFATc1 pathways [69]. Other compounds, such as casticin (*Artemisia annua*), α-mangostin (*Garcinia mangostana*), and resveratrol (from grapes), revealed anti-osteoporosis effects via the NF-κB signaling pathway in different experimental models [70–72]. In TCM formulae, Buqi-Tongluo Decoction inhibited osteoclastogenesis and alleviated bone loss in ovariectomized rats by attenuating NFATc1, MAPK, NF-kappaB signaling [73]. Yougui Yin and its herbal component Cuscuta chinensis revealed to regulate macrophage polarization through the NF-κB/IκBαsignaling pathway [74, 75]. Therefore, the NF-κB signaling pathway is commonly studied to explore the therapeutic prinicples of medicinal plants for osteoporosis treatment.

### “Gut-bone” axis

Recent progress draw attentions to the roles of gut microbiota dysbiosis in the pathogenesis of osteoporosis, showing the reduced microbial diversity and richness, as well as the declined beneficial bacteria and increased pro-inflammatory taxa. The imbalances of “gut-bone”axis impair intestinal barrier function and height systemic inflammatory state [76]. Dysbiosis stimulates T cells to secrete pro-inflammatory cytokines, including TNF-α, IL-6, and IL-17 [77]. These cytokines promote bone loss by upregulating RANKL expression, suppressing OPG production, and thereby enhancing osteoclast differentiation and bone resorption [78]. Additionally, the gut microbiota affects the absorption of essential minerals such as calcium, magnesium, and phosphorus [79].

**Fig. 2:**
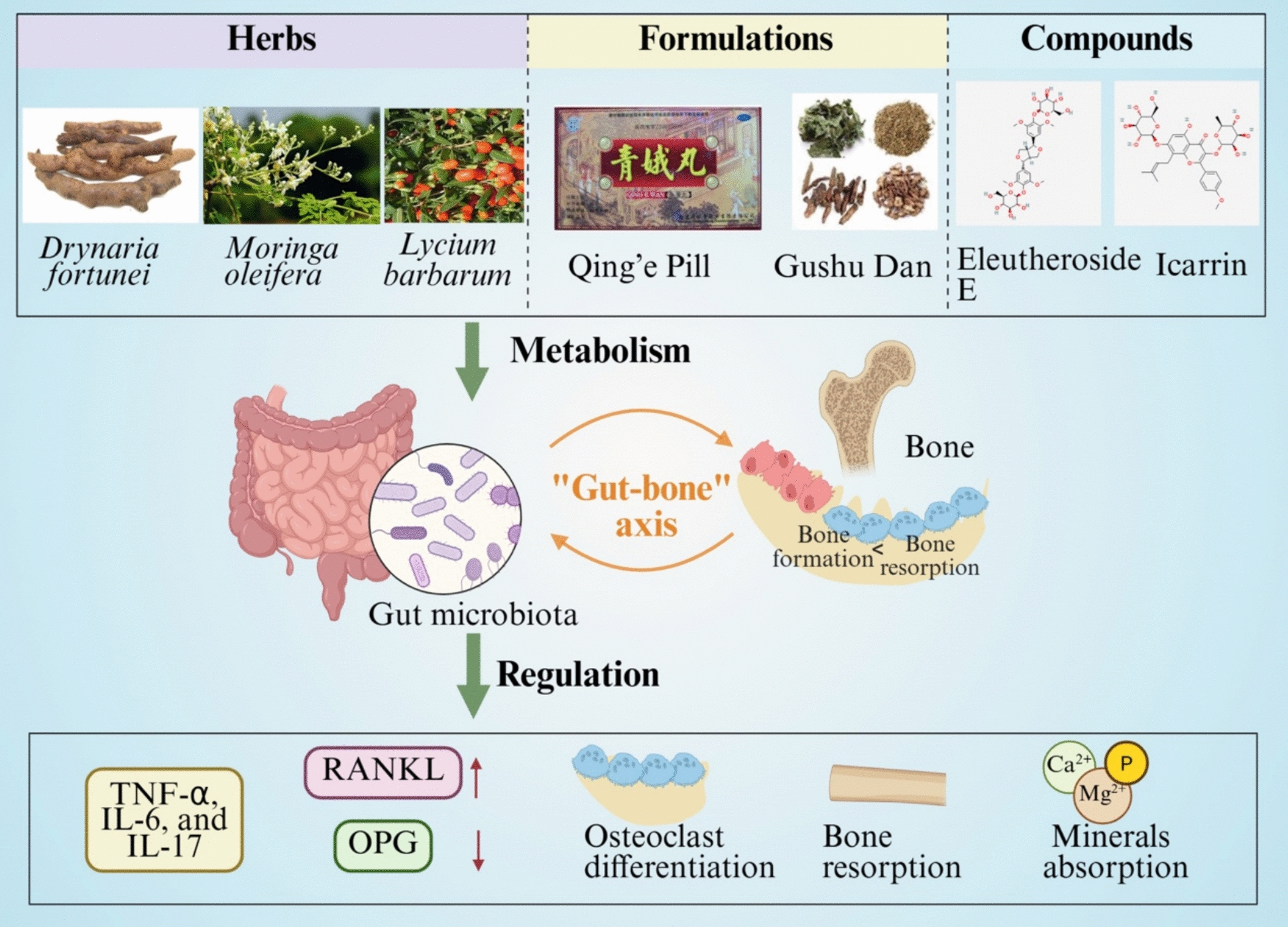
Current understanding of medicinal plants and their active compounds targeted the gut-bone axis for anti-osteoporosis

Evidence suggests that medicinal herbs may modulate the gut microbiome to influence the progression of osteoporosis. For example, Drynariae Rhizoma (*Drynaria fortunei*) alleviated bone loss in OVX rats by reshaping gut microbial composition and metabolic activity, increasing community richness, and restoring specific genera associated with bone formation [80]. Similarly, *Moringa oleifera* leaf extract improved bone mineral density, microstructure, and lipid profiles by regulating gut microbiota composition and the MAPK signaling pathway, thereby enhancing the bone microenvironment [81]. Icarrin, a bioactive compound from *Epimedium brevicornum*, regulated osteoblast differentiation through miR-335-5p/PTEN/IGF-I signaling and modulated gut microbiota composition to improve calcium absorption [82]. Qing’e Pill regulated gut microbiota, increased production of short-chain fatty acids (acetate, propionate, and butyrate) in OVX-induced osteoporosis models, indicating the immunomodulatory and bone-protective properties [83]. In addition, Gushu Dan, *Lycium barbarum*, and Eleutheroside E (from *Acanthopanax senticosus*) also revealed the bioactivities of regulating gut microbiota and related metabolic pathways, such as branched-chain amino acids metabolism [84–87]. These findings collectively support the therapeutic relevance of gut microbiota-targeted TCM interventions in osteoporosis management. The representative diagram summarizes the representative medicinal herbs and TCM formulae and active compounds with the bioactivities of modulating gut microbiota and anti-osteoporosis (Fig. 2).

### Antioxidant properties for bone remodeling

Bone remodeling is highly susceptible to redox imbalances. The accumulation of reactive oxygen species (ROS) elevates mitochondrial metabolic rates and exacerbates oxidative stress within bone cells [88]. Endogenous antioxidant enzymes, such as glutaredoxin 5 (Grx5) and glutathione peroxidase (GPX), have protective effects on osteoblasts. Conversely, the transcription factor FOXO impairs osteogenesis by competing with β-catenin and attenuating Wnt/β-catenin signaling [89–91]. Oxidative stress further exacerbates bone loss by promoting osteoclast activity. ROS stimulates RANKL production and enhances TNF-α secretion under estrogen-deficient conditions, subsequently driving osteoclast differentiation and bone resorption [92, 93]. Additionally, NADPH oxidase-derived ROS, including superoxide anions and hydrogen peroxide, directly facilitate osteoclast differentiation and resorptive activity [94]. Therefore, antioxidant therapy represents a viable therapeutic strategy for limiting pathological bone loss.

Several TCM-derived compounds have demonstrated efficacy in counteracting bone oxidative damage. Luteolin, a common flavonoid, significantly reduces malondialdehyde levels and increases superoxide dismutase activity in OVX mice while improving bone metabolism via the PI3K/AKT pathway [95]. Angelicin enhances antioxidant enzyme levels in osteoblasts through the KAT6A/Nrf2/HO-1 axis, thereby protecting against ROS-induced apoptosis [96]. Icaritin, a natural flavonoid derived from icariin, exhibits osteoprotective effects by stabilizing GPX4 expression and reducing lipid peroxidation in a zebrafish osteoporosis model [97]. Furthermore, *Achyranthis bidentatae* modulates oxidative phosphorylation and transmembrane transport, contributing to redox balance and mitochondrial integrity in bone cells [98, 99]. The effects of TCM Formulas on modulating the gut microbiota have also been reported. These findings collectively underscore the therapeutic potential of TCM in restoring redox homeostasis and protecting against oxidative stress-induced bone deterioration.

### Anti-inflammatory effects

Chronic inflammatory conditions also disrupt bone homeostasis through multiple mechanisms that collectively promote systemic osteoporosis and elevate fracture risk. Pro-inflammatory cytokines, including TNF-α, interleukin-1 (IL-1β), and interleukin-6 (IL-6), directly modulate bone cell activity by enhancing osteoclast differentiation while suppressing osteoblast function—effects that occur independently of RANKL signaling at the preosteoclast level [100]. Estrogen deficiency exacerbates this inflammatory state by upregulating the expression of cytokines and chemokines, which alter the dynamics of bone cell differentiation. The NOD-like receptor protein 3 (NLRP3) inflammasome, abnormally activated in osteoblasts through nuclear factor kappa-B (NF-κB) signaling during estrogen deficiency, significantly contributes to osteoclastic differentiation and bone resorption [101]. Monocytes in postmenopausal osteoporotic women exhibit enhanced survival and osteoclastogenic potential, producing IL-1β and TNF-α that further stimulate peripheral blood mononuclear cells to amplify osteoclast formation [102].

The anti-inflammatory properties of medicinal herbs and their bioactive compounds represent a significant therapeutic approach for managing osteoporosis. For instance, *Rhizoma Drynariae* has emerged as a multifaceted osteoprotective agent, demonstrating the ability to improve bone mineral density and microarchitecture in estrogen-deficient models. Its efficacy is attributed to the modulation of circadian rhythms, lipid metabolism, and immune-inflammatory pathways. Specifically, its total flavonoids exhibit pleiotropic effects, including the suppression of pro-inflammatory signaling and the restoration of bone remodeling balance [61, 103–105]. Additionally, astragaloside IV provides a mechanistic basis for the traditional use of its source herb, primarily through the inhibition of NF-κB signaling and the attenuation of inflammatory cytokine production [66].

### Anti-autophagic effects

Autophagy is a lysosomal-dependent process through which cells degrade unnecessary or dysfunctional components, comprising three main types: chaperone-mediated autophagy, macroautophagy, and microautophagy [106]. This essential cellular mechanism acts as a homeostatic "moderator" by removing damaged macromolecules and replenishing enzymes, cytokines, transcription factors, and adhesion molecules in quiescent stem cells [107]. In bone tissue, autophagy plays dual roles by promoting chondrocyte survival in harsh microenvironments [24] while protecting osteoblasts from oxidative damage through ROS reduction and mitochondrial quality control [108]. During later stages of osteoblast differentiation, autophagy actively participates in bone mineralization, as evidenced by impaired mineralization in autophagy-related 7 (Atg7)- or Beclin-1-deficient models and the presence of apatite crystals in autophagic vacuoles [109]. Autophagy deficiency in osteoblasts may induce endoplasmic reticulum (ER) stress and oxidative damage. In contrast, osteogenic factors such as insulin-like growth factor-I and bone morphogenetic protein-2 enhance osteoblast differentiation by upregulating autophagy-related genes and pathways [108, 110].

Several studies have confirmed the anti-autophagic effects of TCM formulations and their active compounds, suggesting their use for osteoporosis treatment. For example, Si-Zhi Wan decoction exerts anti-osteoclastogenic effects by effectively inhibiting autophagy in osteoclast precursors and downregulating the AMPK signaling pathway [111]. Zhuanggu Zhitong Capsule rebalances bone remodeling and improves bone mineral density by restoring autophagy homeostasis, specifically through targeting the mTOR signaling axis [112]. Similarly, QiangGuYin exerts anti-osteoporotic effects via the AKT/mTOR/autophagy pathway, thereby promoting osteoblast survival and differentiation under stress conditions [113]. At the single compound level, Irisolidone, a natural isoflavone, promotes osteoblast differentiation and accelerates fracture healing by activating the AMPK-Unc-51-like kinase 1-autophagy axis, which enhances autophagic flux and supports osteogenic activity during bone regeneration [114]. Arbutin, a natural hydroquinone glycoside, inhibits autophagy and decreases autophagosome formation, thereby reversing dexamethasone-induced suppression of osteoblast differentiation and mineralization in glucocorticoid-induced osteoporosis models [115]. Furthermore, Jintiange, as a substitute for natural bone tiger, promotes osteogenesis and inhibits osteoblast apoptosis by enhancing autophagic activity through the PI3K/AKT signaling pathway [116].

### Mitochondrial regulation

In skeletal biology, mitochondria play a crucial role in regulating energy metabolism and maintaining bone homeostasis during growth, development, and remodeling [117, 118]. Mitochondrial DNA (mtDNA) encodes essential components of the oxidative phosphorylation (OXPHOS) system required for aerobic respiration and cellular energy production. Clinically, mutations in the *Polymerase Gamma* gene, which is responsible for mtDNA replication, are associated with significant bone loss and increased fracture risk [119–121]. Such mtDNA defects disrupt bone homeostasis through multiple mechanisms: (1) Impairing respiratory chain function and OXPHOS complex assembly, (2) Reducing ATP production and weakening osteoblast mineralization capacity, and (3) Increasing ROS generation due to electron transport chain leakage. Oxidative stress activates pro-osteoclastic signaling pathways (NF-κB and NFATc1) while simultaneously compromising osteoblast function [119–121].

Emerging evidence suggests that TCM holds multifaceted potential for restoring mitochondrial homeostasis as a strategy against osteoporosis. For instance, curcumin, a polyphenolic compound derived from *Curcuma longa*, has been shown to reduce mitochondrial ROS levels, enhance the bioactivities of Complex III and coenzyme Q, and increase mitochondrial membrane potential, thereby attenuating osteoblast apoptosis and mitigating mitochondrial dysfunction [122]. Similarly, Notoginsenoside R1, an active component of *Panax notoginseng*, restores mitochondrial function by suppressing c-Jun N-terminal kinase signaling [123]. The flavonoid naringin prevents OVX-induced bone loss and promotes osteoclast apoptosis through the mitochondria-mediated intrinsic apoptotic pathway [124]. Psoralen, a flavonoid from Psoralea corylifolia, enhances osteogenic differentiation by promoting mitochondrial membrane potential, whose effect is tightly dependent on mitochondrial integrity, as inhibition of the membrane potential abolishes alkaline phosphatase (ALP) activity and calcium deposition [125]. TCM formulations also demonstrate efficacy in targeting mitochondrial pathways. Wen-Shen-Tong-Luo-Zhi-Tong Decoction enhances mitochondrial energy metabolism and osteogenic differentiation in senescent bone marrow stromal cells, exerting protective effects against senile osteoporosis [126]. Additionally, Danggui Buxue Tang significantly improves mitochondrial dynamics in osteoblasts [127]. These findings collectively underscore the role of TCM in preserving mitochondrial function to counteract bone loss.

### Angiogenesis promotion

The coupling between angiogenesis and osteogenesis represents a fundamental biological process in skeletal development and repair [128–130]. In osteoporosis, this vascular-bone unit becomes compromised, characterized by reduced vessel density, impaired vascular function, and endothelial cell damage—features particularly evident in elderly, diabetic, and postmenopausal patients [128–130]. The remodeling process depends on specialized vascular niches that recruit osteoblast and osteoclast precursors. Osteoporotic patients demonstrate significant reductions in key vascular markers, including platelet endothelial cell adhesion molecule-1 (CD31) and vascular endothelial growth factor (VEGF), establishing a clear correlation between impaired vascular supply and decreased bone mineral density [131, 132]. A specialized microvascular subtype termed type H vessel, characterized by the co-expression of CD31 and endomucin (Emcn), has been identified as particularly important for angiogenesis-osteogenesis coupling in trabecular and cortical bone [133]. Both OVX mouse models and clinical comparisons between premenopausal and postmenopausal women reveal significant reductions in type H vessel density associated with bone loss [134]. Consequently, enhancing angiogenesis is a promising therapeutic approach for treating osteoporosis.

With multiple ingradients and multiple targeting approaches, TCM formulae have unqiue advantages to enhance bone repair by simultaneously promoting osteogenesis and angiogenesis. For instance, Qing’e Pill encourages osteogenesis and angiogenesis by upregulating the expression of VEGF and ANG1, thereby enhancing neovascularization and bone repair in vivo [135]. Similarly, Du-Zhong-Wan, primarily composed of *Eucommia ulmoides*, enhances angiogenesis and bone regeneration by promoting type H vessel formation and improving callus strength at fracture sites [136]. The active compound Vitexin, derived from *Panax notoginseng* saponins, promotes trabecular bone repair and enhances osteogenic and angiogenic marker expression, demonstrating dual efficacy in restoring both bone and vasculature [137]. Ginsenoside Rg1 enhances osteoprogenitor survival and VEGF secretion under high glucose conditions, while promoting endothelial cell angiogenesis via Notch-mediated Noggin expression to facilitate osteogenesis–angiogenesis coupling [138]. In diabetic osteoporosis models, Rg1 stimulates type H vessel formation and osteoblast differentiation, thereby improving bone microarchitecture and reinforcing the vascular–osteogenic interface.

**Fig. 3:**
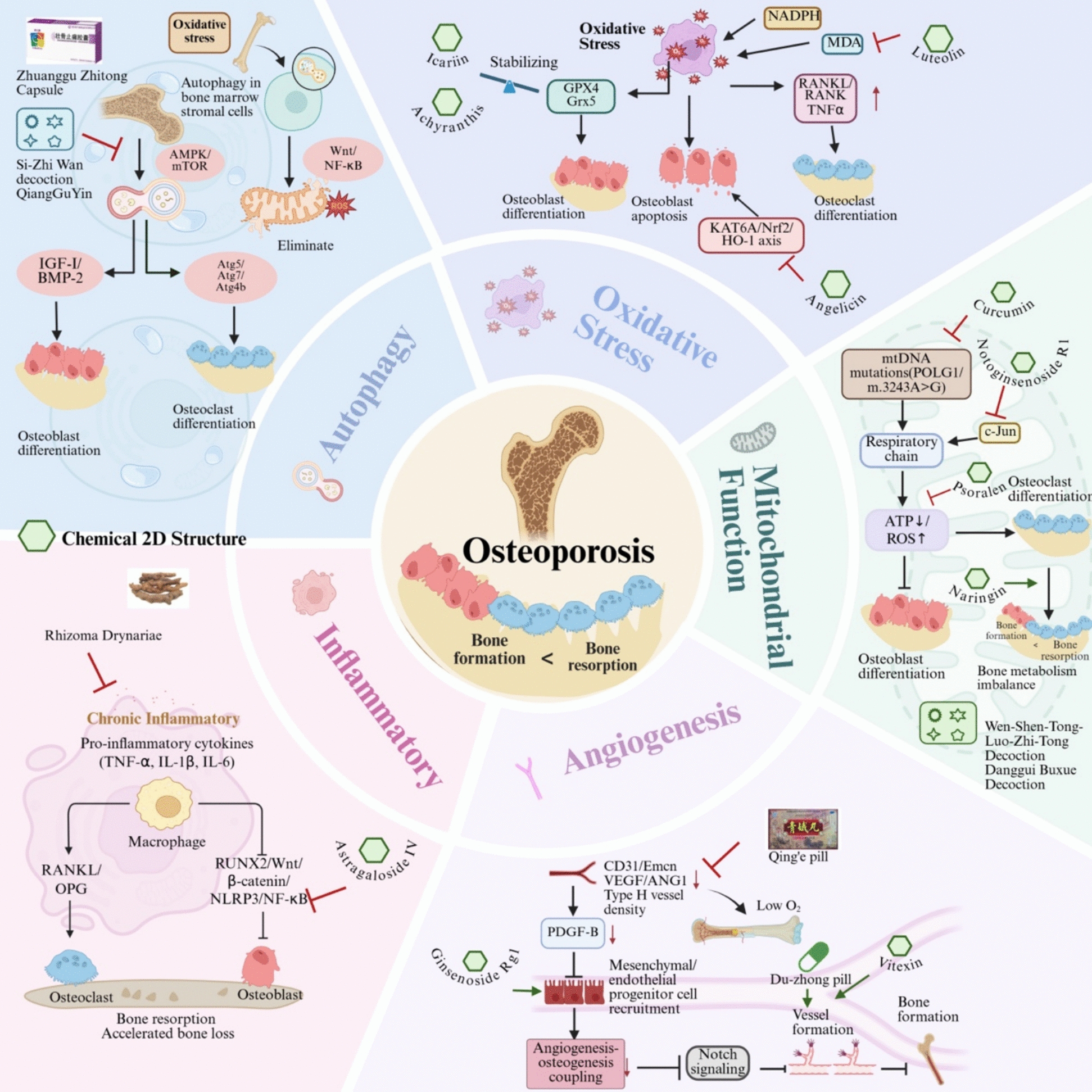
The mechanisms of traditional Chinese herbal medicine formulations, herbal medicine extracts, and active compounds for osteoporosis treatment

In summary, TCM formulae, herbs, and their active compounds exert promising anti-osteoporotic effects through a sophisticated and multi-targeted modulations on the bone remodeling network. Active compounds in TCM formulations could systemically influence key biological axes critical for skeletal homeostasis. The therapeutic principles emerging from these studies highlight several core mechanisms: (1) The promotion of osteogenesis via Wnt/β-catenin and BMP signaling; (2) The suppression of osteoclastogenesis through NF-κB, RANKL, and MAPK pathway inhibition; (3) The restoration of cellular balance by mitigating oxidative stress and mitochondrial dysfunction; (4) The regulation of immune-inflammatory responses; and the modulation of systemic environments via the gut-bone axis and angiogenic coupling. For drug discovery, these findings validate the potential of developing multi-target agents or synergistic formulations that mimic TCM's holistic approach to treat osteoporosis (Fig. 3).

## Clinical studies on efficacy and safety of TCM therapy for primary osteoporosis

Although classical Chinese medical textbook does not explicitly define osteoporosis, its pathogenesis is generally attributed to syndromes such as “Bone Impediment” and “Bone Wilting”, with underlying causes involving *Kidney Deficiency, Liver–Spleen Dysfunction,* and *Depletion of Qi and Blood*, reflecting a complex pathological pattern characterized by “*Root deficiency with Superficial Excess*” and a mixture of deficiency and excess. According to the Prevention and Treatment of Osteoporosis with Traditional Chinese Medicine (2020), clinical interventions mainly focus on promoting bone formation and inhibiting bone resorption[139].

Recent research has expanded this framework by identifying various natural bioactive compounds, such as icariin, constituents from salvia miltiorrhiza, phaseol and glycyrol. Furthermore, Traditional Chinese Medicine formulas and Traditional Chinese Patent Medicines have demonstrated significant clinical efficacy. Evidence suggests that integrating TCM modalities with conventional treatments synergistically prevents disease progression and enhances therapeutic outcomes [140] (Table 3).

**Table 3:** Clinical efficacy and safety of TCM in the treatment of osteoporosis

Formulas	Ingredient	Model	Mechanism	References
Qiangguyin	Astragalus, Honeysuckle, Cinnamon, Eucommia	OVX model	Improved serum level of RUNX2, ALP and estrogen; Activated β-catenin pathway; Regulated AKT/mTOR/autophagy signaling pathway; Promoted osteogenesis and angiogenesis	[33, 113]
Jiangu granules	Epimedium, Eucommia, Achyranthes, Drynaria, Ginseng, alongside	Network pharmacology analysis	Regulated Notch signaling	[45]
Buqi-Tongluo Decoction	Radix Astragali, Radix et Rhizoma Ginseng, Radix Angelicae Sinensis, Rhizoma Chuanxiong, Pheretima	Cell model	Modulated NFATc1/MAPK/NF-κB signaling	[73]
Yougui Yin	Persicae semen, Cinnamomi cortex, Monkshood root, Eucommiae cortex, Lycii fructus, Dioscoreae rhizome, Carthami flos, Corni fructus, Glycyrrhizae radix, Preparata rehmanniae radix	OVX model; Cell model	Regulated NF-κB/IκBα signaling	[74]
Qing’e Pill	Eucommia ulmoides Oliv (Duzhong), Cullen corylifolium (L.). Medik (Buguzhi), Juglans regia L. (Hetaoren), Allium sativum L. (Dasuan)	OVX model	Regulated gut microbiota; Modulated PI3K/Akt signaling; Inhibited MMP-9 synthesis	[83, 144, 145]
Si-Zhi Wan decoction	Eclipta Prostrata L., Fructus Ligustri Lucidi, Angelicae Sinensis Radix, Epimedii Folium (EF)	OVX model	Inhibited osteoclastogenesis; Promoted apoptosis of mature osteoclasts	[111]
Zhuanggu Zhitong Capsule	Psoralea Fruit (Buguzhi), Epimedium (Yinyanghuo), Wolfberry (Gouqizi), Ligustrum lucidum (Nvzhenzi), Drynaria (Gusuibu), Dog's Spine (Gouji), and Achyranthes bidentata (Chuan'niuxi)	OVX model	Regulated autophagy through AMPK/mTOR signaling	[112]

### Traditional Chinese Medicine Formula (TCMF)

TCMF is an integral part of traditional Chinese medicine, encompassing classic prescriptions summarized by physicians throughout history in clinical practice, which include capsules, tablets, granules, pills, and mixtures. The existing evidence supports the efficacy of TCMFs in the clinical management of osteoporosis [141].

Qing’e Formula (QEF) is a TCMF composed of *Eucommia ulmoides Oliv*, *Medik*, *Cullen corylifolium*, *Allium sativum L*., and *Juglans regia L*., with its proportions standardized according to the Chinese Pharmacopoeia. In China, the use of QEF can be traced back over 1000 years to the Song Dynasty. It is traditionally recognized for its functions of tonifying the kidney, strengthening the body, enhancing physical fitness, and improving vitality [142]. A clinical trial involving 60 postmenopausal osteoporosis patients with bone pain demonstrated that QEF treatment alleviated pain in 80% of participants, while also reducing serum levels of IL-6 and TNF-α. It increased the level of IGF-1 [143]. In vivo studies further revealed that QEF improved BMD and biomechanical properties in ovariectomized osteoporotic rats by modulating the PI3K/Akt signaling pathway [144]. Additionally, QEF inhibited the synthesis and secretion of MMP-9 in bone tissue and reduced TRACP activity, contributing to bone protection and remodeling [145].

Zuogui Pill (ZGP) combines with *Rehmanniae Radix Praeparata*, *Rhizoma Dioscoreae*, *Fructus Lycii*, *Fructus Corni*, *Cyathulae Radix*, *Semen Cuscutae*, *Colla Cornus Cervi*, and *Colla Plasti Testutinis* [146]. A multicenter, randomized, double-masked, placebo-controlled trial involving 200 patients demonstrated that short-term treatment with ZGP significantly improved lumbar spine (L1–L4) BMD, visual analogue scale scores for pain, and health-related quality of life (HRQoL), while regulating the coupling balance between bone formation and bone resorption [147]. In addition, a study of 200 osteoporosis patients showed that ZGP treatment markedly alleviated clinical symptoms, improved BMD, inhibited bone conversion with few and mild adverse reactions, such as mild diarrhea [148]. Furthermore, a randomized controlled trial involving 80 postmenopausal osteoporosis patients with *Kidney Yin Deficiency* revealed that Modified ZGP significantly enhanced BMD and improved clinical symptoms, further supporting its therapeutic potential in the management of osteoporosis with *Yin Deficiency* patterns [149]. In ovariectomized animal models, ZGP ameliorated osteoporosis by modulating the gut microbiota [150]. Mechanistically, ZGP promoted osteoblast differentiation and regulated key hub genes, such as let-7f, mTORC1, and Runx2, to suppress autophagy [151]. Through these mechanisms, ZGP contributes to influencing inflammatory responses, osteoclast differentiation, and hormone metabolism, thereby preventing and treating glucocorticoid-induced osteoporosis in rats, and improving bone quality and metabolic balance.

Liuwei Dihuang Decoction (LWDHD) is a classical traditional Chinese formula composed of six herbs: *Radix Rehmanniae, Fructus Corni, Rhizoma Dioscoreae, Rhizoma Alismatis, Cortex Moutan Radicis, and Poria* [152]. A clinical trial involving 120 postmenopausal osteoporosis patients with *Kidney Yin Deficiency* demonstrated that treatment with LWDHD effectively reduced bone turnover markers, including serum procollagen type I N-terminal propeptide (P1NP) and β-isomerized C-terminal telopeptide of type I collagen (β-CTX), increased BMD in the lumbar spine and hip, and significantly improved patients’ quality of life [153]. Furthermore, another study recruited 126 osteoporosis patients with *Liver–Kidney Yin Deficiency*. LWDHD combined with Aclasta significantly increased serum levels of calcium, total vitamin D, bone gamma-carboxyglutamic acid protein, alkaline phosphatase, parathyroid hormone, and β-CTX, while markedly reducing total procollagen type I N-terminal propeptide (P1NP) expression. The combined therapy released pain and improved quality of life with adverse reactions [154]. The HIF-1 signaling pathway and the TNF signaling pathway could be the therapeutic targets of LWDHD [155]. The anti-osteoporosis of LWDHD could be attributed to regulate bone metabolism, inflammatory responses, and cellular proliferation and differentiation in postmenopausal osteoporosis patients associated with *Kidney Yin Deficiency* by upregulating CLCF1 and activating the JAK/STAT signaling pathway [156].

Erzhi Pill is composed of *Herba Ecliptae* and *Fructus Ligustri Lucidi* with the functions of *Tonifying Liver and Kidney Yin*. Erzhi Pill revealed to regulate bone metabolism and alleviate symptoms in osteoporotic patients [157, 158]. Erzhi pill increased BMD, promoted bone formation, and decreased bone resorption in a clinical study involving 96 osteoporosis patients [159]. Network pharmacology revealed that quercetin, apigenin, daidzein, luteolin, ursolic acid, and kaempferol could be key compounds of Erzhi Pill that target multiple signaling pathways for anti-osteoporotic bioactivities, including PI3K-Akt, TNF, and IL-17 signaling pathways [160]. The Erzhi pill demonstrated its promising pharmacological action in promoting osteoblast proliferation and inhibiting osteoclast differentiation for the treatment of osteoporosis [158].

Overall, those classical TCM formulae are valuable therapeutic agents for osteoporosis whose underlying mechanisms remain to be further elucidated.

### Traditional Chinese Patent Medicines (TCPMs)

TCPMs are standardized oral formulations derived from fixed herbal prescriptions with comprehensive clinical trial data to support their application for osteoporosis [161]. Qianggu Capsule is a representative TCPM containing total flavonoids extracted from *Gusuibu* (*Rhizoma Drynariae*) approved by Chinese National Medical Products Administration for the treatment of osteoporosis [162, 163]. Preclinical and clinical studies support its anti-osteoporotic efficacy by increasing BMD and elevating blood levels of alkaline phosphatase, calcium, and osteocalcin [164, 165]. In a clinical trial involving 90 postmenopausal osteoporosis patients, Qianggu Capsule significantly alleviated bone pain, improved muscle strength, reduced the incidence of recurrent fractures, and enhanced overall quality of life, confirming its therapeutic efficacy and safety [166]. Another clinical study of 120 postmenopausal osteoporosis patients showed that Qianggu Capsule significantly reduced both resting and movement-related lumbar pain, while increasing serum levels of osteocalcin, calcitonin, and estradiol, further supporting its favorable clinical profile [167]. A meta-analysis on nine randomized controlled trials (RCTs), involving 806 patients with postmenopausal osteoporosis, demonstrated that Qianggu Capsule significantly increased lumbar spine BMD and reduced bone pain with mild adverse reactions including transient constipation, dry mouth, abdominal discomfort, and chest tightness. Thus, Qianggu Capsule is a safe and effective TCM therapy for postmenopausal osteoporosis [165].

Xianling Gubao Capsule (XLGB) is a composite formulation containing *Longspur epimedium*, *Radix Dipsaci*, *Psoralea corylifolia*, *Radix Rehmanniae*, *Salvia miltiorrhiza*, and *Rhizoma Anemarrhena*, with the functions for *Tonifying Liver and Kidney, Promoting Blood Circulation*, and *Strength Bones* according to the TCM concept [168]. A meta-analysis of 107 RCTs, involving 10,032 participants, found that XLGB demonstrated superior overall clinical effectiveness, significantly improved bone mineral density at the lumbar spine and femoral neck, and increased serum levels of bone Gla protein, with no severe adverse events reported [169]. In another meta-analysis involving eight RCTs, XLGB significantly reduced serum tartrate-resistant acid phosphatase (TRACP) levels and visual analogue scale (VAS) pain scores, indicating inhibition of bone resorption and effective pain relief. Notably, XLGB did not increase the incidence of adverse events compared to conventional treatments, underscoring its favorable safety profile [170].

Mechanistically, XLGB downregulates RANKL mRNA expression while upregulating OPG mRNA, thereby inhibiting osteoclast activity and promoting the differentiation and mineralization in osteoblastic MC3T3-E1 cells [171]. Network pharmacology analyses further suggest that its active components may exert therapeutic effects by modulating key signaling pathways involved in bone homeostasis, including Wnt, TNF, MAPK, and PI3K-Akt [172].

### Nature active compounds

Many active compounds have been identified from medicinal plants with anti-osteoporosis bioactivities with the potentials to be promising drug candidates or healthy food supplements. Several natural active compounds have been clinically tested for osteoporosis treatment. However, current clinical studies on the anti-osteoporosis natural compounds are at premature stages which are predominantly at small-scale clinical trials as healthy food supplements or as adjuvant therapies for complemental treatment.

Icariin is an active flavone glycoside isolated from *Epimedium plants* with anti-osteoporosis activity [173] In a 24-month randomized, double-blind clinical trail involving 360 treatment subjects and 120 control subjects, *Epimedium total flavone* capsule revealed its effects on releasing osteoporosis symptoms, alleviating bone pain and increasing BMD in the osteoporosis patients [174]. Another 24-month randomized double-blind placebo-controlled trial demonstrated that icariin, daidzein and genistein significantly reduced bone loss in postmenopausal women [175]. The major adverse events of icariin include gastrointestinal dysfunction, constipation, tinnitus, and cardiopalmus [174].

*Salvia miltiorrhiza Bunge* named Danshen in Chinese is a commonly used as an anti-osteoporotic medicinal plant in TCM practice [176, 177]. In a clinical trial involving 86 patients with primary osteoporosis, Danshen injection is a standardized agent approved by China National Medical Products Administration and derivated from *Salvia miltiorrhiza Bunge* containing both lipophilic and hydrophilic ingredients, such as Tanshionone I, Tanshinone IIA, Danshensu, Salvianolic acid A, B, C and D, etc. Treatment of Danshen injection significantly increased in BMD and alleviated pain in osteoporosis patients [178]. Tanshinone, a lipophilic compound derived from *Salvia miltiorrhiza*, mitigated the OVX-induced osteoporosis via upregulating the mRNA expression of phosphoglycerate dehydrogenase [179]. Dihydrotanshinone I is a phenanthrenequinone compound isolated from the roots of *Salvia miltiorrhiza Bunge*. Dihydrotanshinone I prevented osteoclast formation and mitigated estrogen-deficiency osteoporosis [180]. *Tanshinone IIA*, a major active ingredient, revealed to be effective for osteoporosis treatment. A single center RCT involving 40 cases of primary osteropososis revealed that *Tanshinone IIA* significant increased in serum PINP levels and decreased β-CTX levels in primary osteoporosis patients [181].

Other nature compounds also revealed robust effects on regulating osteoblast differentiation at the molevular level. For example, Phaseol is a naturally coumenstan compound found in leguminous plants such as licorice, mung beans and soybeans [182–184]. Phaseol was identified as a potent TAK1 inhibitor with dual roles in enhancing osteoblast function and suppressing osteoclast activity [185]. Similarly, Glycyrol is a natural coumestan compound derived from licorice. Glycyrol modulated Syk signaling cascades and mitigated osteoporosis through regulating osteoclastogenesis and osteogenesis [186]. Overall, the active compounds isolated from medicinal plants are important sources of drug discovery for osteoporosis treatment. Clinical trials warrant their effectiveness and saftety in the treatment of osteoporosis.

### Adverse drug effects

The global adoption of TCM has expanded alongside growing attention to its potential adverse effects. In 2020, reports submitted to the China National Adverse Drug Reaction Monitoring Center documented 1.676 million adverse reaction/event cases related to medications on the National Essential Medicines List (2018 Edition). Among these, 88.1% involved chemical drugs and biological products, while 11.9% were associated with traditional Chinese medicines [187].

Further insight into the safety profiles of TCM treatments for osteoporosis comes from specific clinical studies. In one trial involving 93 patients treated with Gukang Capsule, reported side effects included gastrointestinal, hepatobiliary, dermatological, urinary, and respiratory disorders. After discontinuation of the medication and symptomatic management, 25 patients recovered completely and 67 showed clinical improvement, with one case outcome remaining undetermined [188].

Additionally, a meta-analysis on a *Kidney-Tonifying* TCM formula for postmenopausal osteoporosis indicated that adverse effects were primarily mild to moderate gastrointestinal discomfort, such as constipation, diarrhea, nausea, and vomiting [189]. Other reported clinical manifestations include fatigue, anorexia, nausea, upper abdominal pain, and jaundice. These risks may be elevated with long-term or continuous use, particularly among elderly populations [189]. Notably, a case report on *Xianling Gubao* Capsule highlighted a potential risk of drug-induced liver injury [190].

In summary, based on current clinical evidence, most adverse drug reactions associated with TCM for osteoporosis are mild to moderate. The majority of these reactions can be effectively managed by discontinuing the medication and providing appropriate symptomatic care, supporting the overall clinical safety profile of TCM when used properly. However, many adverse reactions are linked to factors such as irrational drug combinations, incorrect dosing, and improper administration. To minimize risks, it is essential to strictly control dosage and treatment duration, remain vigilant regarding contraindications and potential drug interactions, and implement regular patient monitoring in clinical practice.

## Challenges and limitations in the translation of TCM and future actions

TCM treatment for osteoporosis shows great promise, but the transition from traditional theories to new drugs that meet modern evidence-based medicine standards still faces a series of severe challenges and limitations. These problems mainly focus on two aspects: bioavailability and standardized quality control.

### Limited bioavailability and solutions

TCM active ingredients often exhibit inherently low oral bioavailability due to their complex chemical structures and specific physicochemical properties, such as high lipophilicity and poor aqueous solubility, which severely limit their clinical efficacy and scientific translation [191]. For instance, *Icariin* has an oral bioavailability of only about 12% and a short plasma half-life (1.2–3.5 h), which complicates the assessment of its therapeutic effects [192]. Similarly, tanshinone IIA suffers from low water solubility and significant first-pass metabolism, resulting in oral absorption rates as low as 2.1–6.17% [193]. Thus, it is necessary to advance drug delivery systems and modify the formulation techniques.

To address these limitations, advanced drug delivery systems and novel formulation strategies are being developed. Encapsulation within nanocarriers has proven effective in enhancing the water solubility, stability, and transmembrane absorption of TCM compounds [194]. For example, icariin-loaded nanocarriers enable targeted delivery to bone tissue, increasing local concentration and therapeutic efficacy in ovariectomized mice [195]. Similarly, celastrol nanocarriers have been shown to suppress osteoclastogenesis in postmenopausal osteoporosis models [196]. Furthermore, combining TCM with absorption enhancers, such as exogenous snailase, has improved oral bioavailability and anti-osteoporotic efficacy in rats [197]. These innovative pharmaceutical approaches provide promising solutions to bioavailability challenges, supporting the modernization and clinical effectiveness of TCM in osteoporosis management.

### Batch-to-Batch quality consistency evaluation

Batch-to-batch quality consistency is a critical focus in the global evaluation of TCM’s efficacy and safety. Given that the quality of herbal materials is highly influenced by cultivation location, climate, harvest time, and storage conditions, among other factors, it is challenging to ensure the consistency of the final product [198, 199]. Furthermore, the multiple processing steps can induce complex chemical and physical changes in the production of TCM. The relationships between raw material properties, process parameters, and final product quality remain poorly understood due to limited process knowledge [200, 201]. Consequently, establishing robust quality assessment methods is essential.

Chromatographic fingerprinting serves as a reproducible method for characterizing the comprehensive chemical composition of TCM preparations, enabling practical evaluation of batch-to-batch consistency. This approach supports quality assurance through three primary mechanisms: First, by using advanced analytical techniques—such as HPLC, GC–MS, or LC–MS—to establish a representative chemical "fingerprint" of a product, which can be compared against a reference standard to verify stability and uniformity across batches; Second, by providing traceability and guiding process optimization through objective tracking of raw materials from source to finished product; and Third, by correlating fingerprint profiles with specific pharmacological outcomes such as anti-osteoporotic activity to identify key active component groups and advance the scientific basis for TCM modernization [202, 203]. For instance, *Xianling Gubao* Capsule (XLGB), a widely used TCM for osteoporosis treatment, contains a complex mixture derived from six herbal ingredients, posing significant challenges for quality standardization. To address this, an ultra-high-performance liquid chromatography–quadrupole time-of-flight mass spectrometry (UPLC/Q-TOF–MS) method was developed to simultaneously quantify 18 representative components across 34 different XLGB batches [204]. The selection of these quantitative markers was based on multiple criteria, including in vitro anti-osteoporotic activity, in vivo plasma absorption profiles, and pharmacopoeia-specified chemical standards. The resulting data demonstrated strong batch-to-batch consistency for most samples, validating the effectiveness of this advanced fingerprinting strategy in ensuring the quality stability of complex botanical drug products.

### Insufficient high-quality RCT studies

The clinical trials aiming to investigate TCM for osteoporosis have been increasing recently. However, high-quality, large-scale, randomized controlled trials (RCTs) are still lacking, continuously limiting the global acceptance and application of these therapies. Current RCTs often lack rigorous methodological standards, such as prospective registration and blinding, which compromises the reliability of their outcomes. For instance, a systematic review on *Epimedium* in osteoporosis found that while it modulates multiple molecular pathways and the bone microenvironment, the evidence remains insufficient to confirm its efficacy definitively. The included studies were generally limited by inconsistent results and small sample sizes [205]. Similarly, a meta-analysis on Gukang Capsule indicates that the absence of double-blinding, long-term safety data, and standardized outcome measures restricts the evidence supporting its clinical use [206]. Another unignorable limitation is that current RCTs were conducted almost exclusively within Chinese populations, without broader global validation. This narrow demographic scope introduces potential bias and limits the generalizability of the findings. To advance international recognition, it is essential to implement more rigorous trial designs, expand studies to diverse populations, and conduct well-controlled and multi-center RCTs to robustly substantiate the efficacy and safety of TCM in treating osteoporosis.

## Discussions and perspectives

Osteoporosis represents a complex multifactorial disease characterized by the interplay of numerous pathophysiological mechanisms. Epidemiological studies demonstrate a clear correlation between aging populations and the rising prevalence of osteoporosis, with projections indicating that this trend will continue to accelerate [207]. The disease pathogenesis involves an intricate network of risk factors, including hormonal changes, nutritional deficiencies (particularly vitamin D and calcium), genetic predisposition, and lifestyle elements such as physical inactivity and smoking [208]. This complex etiology necessitates therapeutic approaches that can simultaneously address multiple pathological pathways.

TCM has emerged as a particularly promising therapeutic paradigm due to its inherent multi-target nature. With a clinical history spanning millennia, TCM's efficacy in osteoporosis management is now being validated through modern pharmacological research [15]. The identification and characterization of bioactive compounds such as icariin, naringin, and astragaloside IV have provided scientific substantiation for TCM's traditional use. These compounds exhibit pleiotropic effects, targeting oxidative stress through Nrf2/HO-1 activation, inflammation via NF-κB inhibition, and cellular senescence by modulating the STING pathway [66]. Importantly, they also address emerging therapeutic targets, such as the gut-bone axis, with icariin demonstrating significant microbiota-modulating properties [82].

The translation of these findings to clinical practice presents both opportunities and challenges. Current research highlights several lead compounds with exceptional therapeutic potential. Icariin has progressed to Phase II clinical trials (NCT04308382) following robust preclinical results, which showed an 18.7% improvement in BMD [209]. Naringin exhibits superior pharmacokinetic properties with 65% oral bioavailability [65], while astragaloside IV offers novel senotherapeutic effects [66]. However, significant hurdles remain in standardization, with natural variations in active compound content (e.g., a 5–20% variation in icariin content in Epimedium [210] requiring stringent quality control measures.

Future research should prioritize three key areas: (1) mechanistic studies to elucidate compound interactions with emerging targets like Wnt10b [37] and cellular senescence pathways [66]; (2) formulation development to enhance bioavailability, as demonstrated by nanoparticle-encapsulated icariin achieving 38% absorption [209]; and (3) comprehensive clinical validation through well-designed trials with fracture prevention endpoints. Particularly, emphasis should be placed on investigating synergistic combinations, such as icariin with low-dose bisphosphonates, which have shown additive effects in preclinical models.

## Conclusion

The integration of traditional medical knowledge with contemporary scientific investigation has positioned TCM as a valuable source of novel therapies for osteoporosis. The multi-target actions of compounds like icariin, naringin, and astragaloside IV address the complex pathogenesis of osteoporosis more comprehensively than single-target conventional drugs. While challenges in standardization and bioavailability persist, ongoing advances in pharmaceutical technology and quality control are progressively overcoming these limitations. With continued research focused on mechanistic elucidation, formulation optimization, and clinical validation, TCM-derived compounds have the potential to revolutionize osteoporosis management by providing safer, more comprehensive treatment options tailored to the multifaceted nature of bone loss in aging populations.

## Data Availability

No data was used for the research described in the article.

## Abbreviations

TCM: Traditional Chinese Medicine
BMD: Bone mineral density
NF-κB: Nuclear factor kappa-light-chain-enhancer of activated B cells
RUNX2: RUNX family transcription factor 2
RANKL: Nuclear factor-κΒ ligand
M-CSF: Macrophage colony-stimulating factor
Wnt: Wingless and INT-
TGF-β: Transforming growth factor β
ROS: Reactive oxygen species
RNS: Reactive nitrogen species
SOD: Superoxide dismutase
TNFα: Tumor necrosis factor-alpha
OPG: Osteoprotegerin
IL-1β: Interleukin-1β, IL-6, interleukin-6
CCR2: C–C Motif Chemokine Receptor 2
NLRP3: NOD-like receptor protein 3
NF-κB: Nuclear factor kappa-B
DKK1: Dickkopf-1
BMSCs: Bone marrow stromal cells
Atg7: Autophagy related 7
PDGF-B: Platelet-derived growth factor-B
ALP: Alkaline phosphatase
OVX: Ovariectomized
AMPK: AMP-activated protein kinase
JTF: Jin-tian-Ge
mtDNA: Mitochondrial DNA
VEGF: Vascular growth factor
RCT: Randomize controlled trials
